# Supplementary figures and images for: User experience with pregnancy tracker mobile apps: Findings from comment-based qualitative study
Source: PLoS One. 2025 Feb 10;20(2):e0318012. doi: 10.1371/journal.pone.0318012 (PMC11809848; doi:10.1371/journal.pone.0318012)

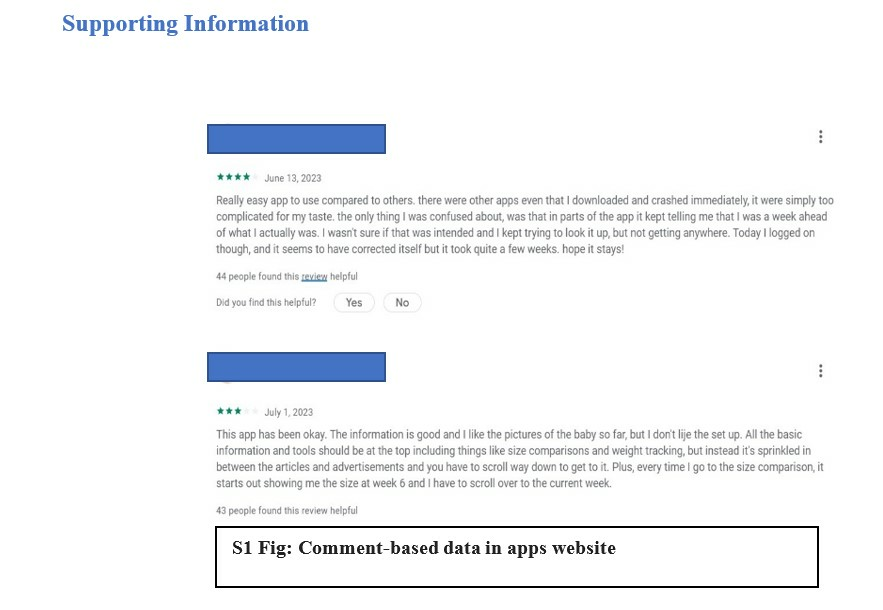

Supplement: S1 Fig — (TIF) [file pone.0318012.s001.tif]
