# Supplementary material for: User experience with pregnancy tracker mobile apps: Findings from comment-based qualitative study
Source: PLoS One. 2025 Feb 10;20(2):e0318012. doi: 10.1371/journal.pone.0318012 (PMC11809848; doi:10.1371/journal.pone.0318012)
Supplement: S1 Table — (DOCX) [file pone.0318012.s002.docx]

**S1 table:** **Standards for reporting Qualitative Research (SRQR) guideline**

| **No** | **Topic** | **Items** |
| --- | --- | --- |
| **Title and abstract** | | |
| S1 | Title | **User Experience with Pregnancy Tracker Mobile Apps: Findings from Comment-based Qualitative Study** |
| S2 | Abstract | **Background**  Worldwide, millions of pregnant women use pregnancy-related apps to monitor their baby’s growth and development. While most of the apps are user-friendly, not all of them are equally appealing. This study aimed to explore the user experience (UX) of pregnancy tracker mobile apps used by pregnant women.  **Methods**  This study explored the dynamics between users' experiences and multifaceted dimensions of advanced features, high-quality materials and information, strict privacy policies, problem-solving abilities, and the usefulness of app features and contents. This study applied reviewers' comment-based qualitative study, accessing crowdsourced data gathered from different pregnancy tracker app websites. A thematic and content analysis approach was used.  **Results**  This study found that when users are satisfied with using advanced content and features, it aligns with their perceived self-righteousness and rationality, and reflects their cultural values and expectations of using the apps. Conversely, when users encounter challenges such as erroneous baby size comparison and app updating issues, they perceive these as disadvantages of the apps utilised.  Moreover, the study sheds light on the specific desires of pregnant women, highlighting their expectations for content that addresses their physical and mental well-being, as well as their unborn babies. The desire for free access reflects the cultural emphasis on cost-effectiveness, while the willingness to invest financially in enhanced experiences demonstrates the recognition of the value and potential benefits of improved content and features. This study also provides valuable insights into the complex relationship between users' experiences, cultural values, advanced features, high-quality materials and information, privacy policies, problem-solving abilities, and relevant content in creating positive app experiences that align with users' cultural expectations and needs.  **Conclusion**  This study provides essential insights into the user experience and underscores the importance of a user-centric design approach for developers. By capturing the current landscape of these digital tools, can provide valuable feedback for enhancing existing applications and guide the development of future iterations, ensuring the diverse preferences and expectations of pregnant women worldwide. |
| **Introduction** | | |
| S3 | Problem formulation | Hundreds of apps are regularly available, but very few of them were developed following rigorous scientific procedures. So, it is necessary to ensure the quality of these apps to give pregnant women a better experience of their pregnancy. However, limited studies have been conducted to explore the quality of the existing pregnancy apps through user opinions. |
| S4 | Purpose or Research Question | This study aims to explore the user experience (UX) of pregnancy tracker mobile apps on Android and iOS used by pregnant women |
| **Methods** | | |
| S5 | Qualitative approach and research paradigm | This study adopted comment-based qualitative study approach, accessing crowdsourced data through customer review feedback, and comments. A thematic and content analysis approach was used for analysing the data. |
| S6 | Researcher characteristics and reflexivity | Two researchers independently analysed the coded data to find out the similarities and differences until a consensus had been reached. Later, we thematically presented the results and interpreted the study results |
| S7 | Context | This study was conducted on the [Google](http://Google) platform. Data was obtained from different pregnancy tracker app websites. |
| S8 | Sampling strategy | Purposive sampling strategy |
| S9 | Ethical issues about human subjects | This study was conducted for partial fulfillment of the executive certificate course on population health informatics. The exam board members oversee the ethical and research issues and approve the proposal and thesis by the BRAC James P Grant School of Public Health, BRAC University. This research does not involve human participation. Therefore, we do not require ethical approval. Moreover, we extracted data from public websites, and for the secondary data, ethical approval and informed consent were not needed. We keep the user’s name and app's name anonymous and respect their privacy while analysing the data. |
| S10 | Data collection methods | We reviewed each user-review comment by visiting the websites of each selected app. Data were extracted from the websites of the ten apps through the Google Play Store (Android) and App Store (iOS). |
| S11 | Data collection instruments and technologies | The comments were selected based on the relevance of the research objectives. We filtered out the irrelevant comments according to the research objectives and short comments that were unable to express the meaning of the objectives as well. |
| S12 | Units of study | Ten apps were included in this study as per the inclusion and exclusion criteria |
| S13 | Data processing | Data we processing using the matrix table |
| S14 | Data analysis | Data were analysis using thematic analysis |
| S15 | Techniques to enhance trustworthiness | Rechecked by the other co-authored and analysed the data. The researcher conducted several online meetings to understand the true meaning of the data |
| **Results/findings** | | |
| S16 | Synthesis and interpretation | The model is generated based on the findings |
| S17 | Links to empirical data | We evaluate the data based on the review comments, ratings and emoji’s they used |
| **Discussion** | | |
| S18 | Integration with prior work, implications, transferability, and contribution to the field | This study found, that users' positive experiences depend on the usefulness of the features and contents of the apps. Advanced features, quality information and materials, strict privacy, and the ability to solve problems make an app appealing to users. Moreover, users' negative experiences depend on encountering update-related issues, problems with functionality and visualisation, technological glitches, the sending of repeated articles, scary posts from peers, gendered issues, and misinformation. Additionally, users' expectations of apps are based on their advanced features and content, such as non-invasive testing, IVF-embryo transfer and visual representation of twin babies and scientifically sound articles. Furthermore, users recommend the app to others when they find fewer advertisements, birth plans and monitoring growth charts, which help expectant mothers establish a spiritual connection with their unborn baby. Finally, pregnancy apps mainly provide desirable features such as data storage, web functionality, personalized tools, and social media integration. This study provides important insights and explains a wide range of thoughts and feelings, including annoyance, concerns, expectations, and usefulness, of pregnancy tracker apps shared by a group of users. Interestingly, useful features and content of the apps impact the users’ overall satisfaction and positive experiences. |
| S19 | Limitations | The data obtained from online platforms may not be representative of all users. Those who leave reviews on app websites might have extreme opinions, and the digital ethnography approach may miss the perspectives of those who do not engage in online discussions. |
| **Other** | | |
| S20 | Conflicts of interest | No conflict of interest among the authors |
| S21 | Funding | No funding was received to conduct this study |
