# Supplementary material for: User experience with pregnancy tracker mobile apps: Findings from comment-based qualitative study
Source: PLoS One. 2025 Feb 10;20(2):e0318012. doi: 10.1371/journal.pone.0318012 (PMC11809848; doi:10.1371/journal.pone.0318012)
Supplement: Attachment — Submitted filename: Response to reviewers.docx [file pone.0318012.s003.docx]

**Response to the reviewer's comments**

We first express our gratitude to the editors and anonymous reviewers for providing your feedback and suggestions, which really enrich the manuscript. We try to address all your comments and the following section provides point-point response to each comment.

**Point-by-point response to the Comments:**

**Editor’s comments:**

**Comment 1.** Please ensure that your manuscript meets PLOS ONE's style requirements, including those for file naming. The PLOS ONE style templates can be found at

[https://journals.plos.org/plosone/s/file?id=wjVg/PLOSOne_formatting_sample_main_body.pdf](https://track.editorialmanager.com/CL0/https:%2F%2Fjournals.plos.org%2Fplosone%2Fs%2Ffile%3Fid=wjVg%2FPLOSOne_formatting_sample_main_body.pdf/1/010f0192de42d82c-d0ab795a-7977-4878-9da0-bd35b349e2ed-000000/wiytoYOmdQSu03isi3JDQD3eHrYYL_TRDcQg2p4j2Dw=183) and

[https://journals.plos.org/plosone/s/file?id=ba62/PLOSOne_formatting_sample_title_authors_affiliations.pdf](https://track.editorialmanager.com/CL0/https:%2F%2Fjournals.plos.org%2Fplosone%2Fs%2Ffile%3Fid=ba62%2FPLOSOne_formatting_sample_title_authors_affiliations.pdf/1/010f0192de42d82c-d0ab795a-7977-4878-9da0-bd35b349e2ed-000000/GiAvxTO66P0qoh7jZCdpUzBzlHsrVHooQtz62FSTzHM=183)

**Reply:** Thank you for your kind suggestion. We have gone through the PLOS ONE formatting and authors affiliation sample. Based on this we have revised the manuscript.

**Comment 2.** In your Methods section, please include additional information about your dataset and ensure that you have included a statement specifying whether the collection and analysis method complied with the terms and conditions for the source of the data.

**Reply:** Thank you for your suggestions.

We have added the additional information in the methodology section

“Data used in this study were extracted from secondary sources which are available in the public domain. These include pregnancy apps used by the general user on the Google platform.”

“The research conducted does not involve human participation and relies on third-party data sourced from public websites. Since the data is secondary, ethical approval and informed consent are not required. The study ensures the anonymity of users and the application involved, thereby respecting privacy during data analysis. Additionally, as no user identification is utilized, permission for data use is not necessary.”

**Comment 3.** Thank you for stating the following in the Acknowledgments Section of your manuscript:

“This study was conducted for partial fulfilment of the executive certificate course on population health informatics. The course program is funded by the Open Society University Network. We thank BRAC James P Grant School of Public Health, BRAC University and icddr,b for their institutional support. We especially thank the core donors of icddr,b the governments of Bangladesh, and Canada for their support.We note that you have provided additional information within the Acknowledgements Section that is not currently declared in your Funding Statement. Please note that funding information should not appear in the Acknowledgments section or other areas of your manuscript. We will only publish funding information present in the Funding Statement section of the online submission form.”

Please remove any funding-related text from the manuscript and let us know how you would like to update your Funding Statement. Currently, your Funding Statement reads as follows:

“The author(s) received no specific funding for this work.”

Please include your amended statements within your cover letter; we will change the online submission form on your behalf.

**Reply:** Thank you for your suggestion. We revise the acknowledgment and funding statements. Given in the below:

**“Acknowledgments**

This study was conducted for partial fulfilment of the executive certificate course on “Population Health Informatics. We thank BRAC James P Grant School of Public Health, BRAC University and icddr,b for their institutional support. We especially thank the core donors of icddr,b the governments of Bangladesh, and Canada for their support.

**Funding statements**

“The author(s) received no specific funding for this work.”

**Comment 4.** For studies involving third-party data, we encourage authors to share any data specific to their analyses that they can legally distribute. PLOS recognizes, however, that authors may be using third-party data they do not have the rights to share. When third-party data cannot be publicly shared, authors must provide all information necessary for interested researchers to apply to gain access to the data. ([https://journals.plos.org/plosone/s/data-availability#loc-acceptable-data-access-restrictions](https://track.editorialmanager.com/CL0/https:%2F%2Fjournals.plos.org%2Fplosone%2Fs%2Fdata-availability%23loc-acceptable-data-access-restrictions/1/010f0192de42d82c-d0ab795a-7977-4878-9da0-bd35b349e2ed-000000/QIVpuqDh6xVaOJdAJ2Df5aML04E8aqB-5lt8BF2Mjqc=183))

For any third-party data that the authors cannot legally distribute, they should include the following information in their Data Availability Statement upon submission:

     1) A description of the data set and the third-party source

     2) If applicable, verification of permission to use the data set

     3) Confirmation of whether the authors received any special privileges in accessing the data that other researchers would not have

     4) All necessary contact information others would need to apply to gain access to the data

**Reply:** Thank you for your comments. We revised the data availability information as like below:

**Data availability**

In this study, we have used open-acess for third-party data and data available in the public domain below mentioned pregnancy tracker apps websites. The URLs of the apps are given below:

1. <https://play.google.com/store/apps/details?id=com.hp.pregnancy.lite&pli=1>
2. <https://apps.apple.com/us/app/pregnancy-tracker-app/id505864483>
3. <https://apps.apple.com/us/app/pregnancy-baby-tracker-wte/id289560144>
4. <https://apps.apple.com/us/app/pregnancy-tracker-babycenter/id386022579>
5. <https://play.google.com/store/apps/details?id=com.ovuline.pregnancy>
6. <https://apps.apple.com/us/app/ovia-pregnancy-baby-tracker/id719135369>
7. <https://apps.apple.com/us/app/pregnancy-tracker-preglife/id390427747>
8. https://play.google.com/store/apps/details?id=androidgecko.com.himommy
9. <https://apps.apple.com/us/app/himommy-pregnancy-tracker-app/id1275040830>
10. https://play.google.com/store/apps/details?id=com.babyalbum.hellobelly

As the data are open access to all, we have provided data sources and added them in data availability list.

**Additional Editor Comments:**

The manuscript provided an interesting content analysis. However, there are multiple writing related issue raised by reviewer including adequate references.
The authors need to work on improving the writing and flow of the analysis as expressed in the reviews.


[Note: HTML markup is below. Please do not edit.]

**Reply:** Thank you for your inspiring comment. We try to improve the writing and flow of the analysis.

**Review Comments to the Author**

Reviewer #1: PONE-D-24-35703
User Experience with Pregnancy Tracker Mobile Apps: Findings from Comment-based Qualitative Study
Thank you for the opportunity to review this manuscript. The authors present findings from a comment-based qualitative study where they evaluated user experience with pregnancy tracker mobile apps. This manuscript deals with an important issue in e-Health/mobile app user perception, usage, and interaction. Overall, they find evidence to support positive user experiences, but also negative experiences of using the different apps.
I enjoyed reading the manuscript; however, there were some sections that were difficult to interpret and that I suggest should be re-written for better reader comprehension (see specific areas below). Similarly, there are some spelling and grammar mistakes that overall do not affect the understanding of the manuscript, but that should be addressed to meet a satisfactory standard for a journal publication.
I am proving here some comments that could potentially improve the understanding of the paper:

**Reply:** Thank you for your encouraging remarks and feedback.

**Comments and response to the reviewer**

**Abstract:**
**Comment:** The first 3 lines in the results section should go in the methods section “the study explores the dynamics between ….” as it mentions the methodology.

**Reply:** Thank you for your kind suggestion. I have added the first three lines of results in the methodology.

“This study explored the dynamics between users' experiences and multifaceted dimensions of advanced features, high-quality materials and information, strict privacy policies, problem-solving abilities, and the usefulness of app features and content. This study applied reviewers' comment-based qualitative study, accessing crowd-sourced data gathered from different pregnancy tracker app websites. A thematic and content analysis approach was used.”

**Comment:** Line 44 in the results section seems to be incomplete: “When users are satisfied with using advanced content and features that align with their perceived self-righteousness and rationality, mirroring the cultural values and expectations”. I suggest that it is re-revised and re-written.

**Reply:** Thank you for your comments. I have revised the mentioned result as per below:

“When users are satisfied with using advanced content and features that align with their perceived self-righteousness and rationality, and reflect their cultural values and expectations to use the apps.”

**Main manuscript:**

**Background:**

**Comment:** lines 68-69. The reference provided is 10 years old and the relative number of pregnancies would be inaccurate. Please update accordingly.

**Reply:** Thank you for your encouraging suggestion. I have revised the Introduction like:

“Annually, unwanted pregnancies totaled 121·0 million in 2015–19, which translates to a global rate of 64 unplanned pregnancies per 1000 women aged 15–49” with an updated reference published in 2020.

**Comment:** Similarly, the statistics reported on lines 71-72 could be updated to represent more current data on maternal deaths.

**Reply:** Thank you for your suggestion.

I have tried to search for updated data, however, I do not find the updated information. Therefore, I keep the information on maternal death. Moreover, I check the overview of the WHO, they also use the same information. Which is updated on April 26, 2024. I add the reference of WHO with the previous information. The link is given below: https://www.who.int/news-room/fact-sheets/detail/maternal-mortality.

**Comment:** Lines 72-75: the sentence: “Some of the factors responsible for adverse maternal health outcomes, though not limited to the unavailability or inaccessibility of antenatal care [3], inadequate maternal nutrition [4], low education status, and lack of health literacy among women, all of which affect their health-seeking behaviour during pregnancy “ is confusing. Please re-write it.

**Reply:** Thank you for your kind suggestion. We have revised the information.

“Unavailability or inaccessibility of antenatal care, inadequate maternal nutrition, low educational status, and lack of health literacy among women significantly influence women’s health-seeking behaviour during pregnancy”.

**Comment:** Line 77: do you mean women seek professional advice? I would not think that they use a pregnancy to seek a consultation with a health professional, as most of these free apps do not provide that service.

**Reply:** Thank you for your question. We have revised the information and given to the below:

“expecting quality content from health expert”

**Comment:** Line 85: reference is from 2013, please update it.

**Reply:** Thank you for your suggestion we have updated the references as per your suggestion. The updated references are given below:

- Pierre-Antoine Fougerouse, M.Y., Guillaume Marchand, Oliver O Aalami, A Cross-Sectional Study of Prominent US Mobile Health Applications: Evaluating the Current Landscape. AMIA Annu Symp Proc, 2018.
- research2guidance. The mHealth apps market is getting crowded. mHealth App Economics 2017 November7, 2024]; Available from: <https://research2guidance.com/mhealth-app-market-getting-crowded-259000-mhealth-apps-now/>.

**Comment:** Line 89: please provide context for this sentence: “The benefits of mobile apps were greater than the challenges”

**Reply:** Thank you kind suggestion. We have added the context. Given in the below:

Studies found that the benefits of mobile apps were greater than the challenges in terms of personalised care like self-management, personalised exercise and monitoring, and helping friends and family avoid harm to dealing with the stroke of elderly people

**Comment:** Lines 105-109: please provide a reference(s) to support these statements.

**Reply:** Thank you for your comments. We have added the references to support the statements. Though two articles were published in 2013, however, it has the strength to support the manuscript. Therefore, we added the references for 2013. The references are given in the below:

- Sandra L. Neate, J.C.R., Craig Hassed, Richard Chambers, Sherelle Connaughton, Nupur Nag, A qualitative analysis of free text comments of participants from a massive open online mindfulness course. Front. Public Health, 2022. 10.
- Schutz, R.W., Qualitative Research: Comments and Controversies. 2013. 60(1): p. 30-35.
- Natalie Henrich, B.J.H., Web news readers comments: Towards developing a methodology for using on-line comments in social inquiry. Journal of Media and Communication Studies, 2013. 5(1).
- Shakeela Ibrahim, M.M.H.A., Ethnographic Approaches to Remote Digital Culture. Journal of Peace, Development and Communication, 2024. 8(2).
- Alessandro Murgia, M.O., Parastou Tourani, Bram Adams, Serge Demeyer, An exploratory qualitative and quantitative analysis of emotions in issue report comments of open source systems. Empir Software Eng, 2018. 23: p. 532-564.
- Noel John Veigas, R.D.S., Dasharathraj K. Shetty, Tojo Thomas, Shreepathy Ranga Bhatta, Nikita Panwar, A Comprehensive Analysis of the User Experience in Digital Platforms Concerning the Practice of Nudging User Behaviour. MDPI, 2023. 59(1).
- William Housley, B.D., Karen Henwood, Robin James Smith, Qualitative methods and data in digital societies. Qualitative Research, 2017. 17(6): p. 607-609.

**Comment:** Line 116: the sentence “comment-based qualitative can explore…” is missing the word ‘studies’. I suggest rephrasing to “comment-based qualitative studies can explore…”

**Reply:** Thank you for your suggestion and please consider our unintentional mistakes.

We have added the missing word “studies” in the revised version.

**Methods:**
**Comment:** Line 136. Data are plural. Please correct the sentence to ‘data were obtained…”

**Reply:** Thank you for your suggestion. We revised the text and please consider our unintentional mistake**.**

**Comment:** Line 146: these findings are part of the results, and as such, should go into the results section of the manuscript. The inclusion and exclusion criteria should precede this.

**Reply:** Thank you for your kind suggestion. We have added the mentioned findings in the results section instead of methodology.

**Comment:** Line 172: it should read ‘to analyse’

**Reply:** Thank you for your kind suggestion. We have revised the text ‘to analyse’ as per your suggestion. Please consider our unintentional mistakes.

**Comment:** Line 183: it should read ‘how data are perceived and analysed”

**Reply:** Thank you for your kind suggestion. We have revised the text as per your suggestion.

**Results:**
**Comment:** Line 203: a short description of table 2 would be useful for the reader.

**Reply:** Thank you for your kind suggestion. We have added about the description of the pregnancy tacker app included in the study in table 2.

“The table 2, consists of the name of the apps, compatibility, name of developers, privacy issues included or not, description of the apps, features and contents, years of starting services, total rating, content rating, number of reviewers, and number of downloads.”

**Comment:** Lines 222-223: consider removing or rephrasing.

**Reply:** Thank you for your kind suggestion. We have rephrased the text as per your suggestion. The text are given in the below:

“Pregnant women often enjoy visualizing their baby's growth, and the app enhances this experience by comparing the baby's size to various fruits and animals. For example, it features playful comparisons like a water bear in the first month, a pygmy seahorse in the second month, and a cardinal tetra fish in the third month, making the journey more engaging and fun.”

**Comment:** Line 251: but the free apps may not ‘offer’ it?

**Reply:** Thank you for your suggestion. We have added the text ‘offer’ instead of contain.

**Comment:** Line 284-288: I do not understand these sentences and how they relate to app issues.

**Reply:** Thank you for your kind suggestion. We have rephrased the text to make understandable.

“On the other hand, users initially tested through the apps using required information and found positive pregnancy results, however, when they found false-negative results based on confirmatory pregnancy tests that impacted their mental stress. A pregnant mother commented,

*“It broke my heart! when I found I am not pregnant after the confirmatory test.” (A user of pregnancy tracker apps on iOS phone)*

**Discussion:**
**Comment:** Lines 411-414. How was this direct link established? These results should be introduced in the result section. No new information/findings should be presented in the discussion section.

**Reply:** Thank you for your suggestion, we have rephrased the text.

“Interestingly, useful feature and content of the apps impacted on the users’ overall satisfaction and positive experiences, sometime it motivates others to use apps.”

**Comment:** Overall, the discussion should not introduce new information that has not been mentioned before. Please add these new findings to the results section.

**Reply:** Thank you for your suggestion. We have revised the discussion section accordingly which is not mentioned in result section.

**Strengths and limitations:**

**Comment:** Line 476-476. Please review this sentence and use academic language "Therefore, we have to review it again and again to get a realistic meaning of the comments"

**Reply:** Thank you for your kind suggestion. We have rephrased the text.

“Therefore, to get a realistic meaning of the comments study team conducted review of the comments repeatedly.”

**Reviewer #2:** Thank you for allowing me to review the manuscript entitled 'User experience with pregnancy tracker mobile apps: Findings from a comment-based qualitative study. The research is an interesting topic and has merit. However, there are some major concerns with the manuscript as it currently presents. Examples of such are outlined below:

**Reply:** Thank you for your encouraging feedback and review.

**Comment:** Overall, the quality of writing is waivers in academic tone. For example, the consistent use of 'we' and the overuse of 'firstly', 'secondly', 'thirdly'... For a more academic tone, I would encourage avoidance of first-person language and, rather than listing points in so many of your sections, aim for a more fluent 'storyline' approach.

**Reply:** Thank you for your kind suggestion. The first-person tone is omitted. However, we keep few as from a qualitative social science perspective it is conventional to use the first person.

**Comment:** I would also suggest a review of language flow and cohesion across the entire manuscript, as there were many instances of disjointed and confusing sentence structure. One example is in the Results section (p10, line 219): "Pregnant mothers are those who use the pregnancy tracker apps and express appreciation...". Pregnant mothers are those who are with child, regardless of their pregnancy tracker app use and appreciation.

**Reply:** Thank you for your kind suggestion. We have revised the text as like below:

“Pregnant mothers express their appreciation for the ease of app use and high-quality photos of the uterus.”

**Comment:** A second example (p11, line 239-240) "By using the app, they can guess the conditions of the baby and mother...". I would suggest reviewing this to say, "By using the app, they may gain insights into how the pregnancy is developing...", rather than imply that it is a guess. However, I do think the whole manuscript might benefit from proofreading revision.

**Reply:** Thank you for your kind suggestion. The text is revised and given into the below:

**“**By using the app, they may main insights into how the pregnancy is developing and encourage the mother to lead a healthy lifestyle.”

**Comment:** The abstract information doesn't seem to completely align with the abstract headings. For example, the first sentence under Abstract Results, should be in the Abstract Methods section. I also think the Abstract Conclusion could be more focused.

**Reply:** Thank you for your suggestion. The first line of the result section replaced to the method section and the conclusion has also been revised as per your suggestion. The conclusion is given below:

“This study provides essential insights into the user experiences and underscores the importance of a user-centric design approach for developers. By capturing the current landscape of these digital tools, can provide valuable feedback for the enhancement of existing applications and guide the development of future iterations, ensuring the diverse preferences and expectations of pregnant women worldwide.”

**Comment:** In the Background section, there are non-formatted (line 68) and/or missing citations throughout (particularly the fourth paragraph [lines 106-123]). I would also encourage you to view pregnancy app reviews by Hayman et al. (2021, 2022) and also the pregnancy influencer study by Hayman et al. (2023), as they may be useful to consider within your background and/or discussion sections.

**Reply:** Thank you for your suggestion we have revised line 68 and also revised lines 106-123. Moreover, we also added references to Hayman et al. in the background and discussion.

**Comment:** The Methods section does not clearly articulate how many and who conducted the search and inclusion/exclusion screening, or data filtering - or if it was done independently. There were also shifts in language tense (past/present/future) that should be addressed throughout this section in particular. Also, a more digestible discussion of the thematic approach (with citations) would be useful to strengthen this section.

**Reply:** Thank you for your suggestion. We have added the number of people who conducted the inclusion and exclusion screening in the methodology section. We also corrected the tense and added discussion about the thematic approach with citations.

**Comment:** Within the Results there are a few instances such as "...baby's size compared with the fruits and animals with fun stuff like..." (p10, line 221-222). The use of "fun stuff" (and similar) is not academic language. Please review the use of language to be highly academic tone.

**Reply:** Thank you for your suggestion. We have revised the text as per your suggestions.

**Comment:** With the Results, under each thematic header, it might be worth structuring the details in a more logically flowing manner. In a few of the paragraphs, the focus seems to shift quite abruptly. A key example of this issue is on page 12 (line 275-293) where the focus starts on the issues caused by app updates, and then jumps to false-positive/negative experiences, then back to technical difficulties with imagery used. Perhaps using third-level headers might be helpful in further structuring the thematic focus points to flow more logically.

**Reply:** Thank you for your suggestion. We have reorganized the results and kept similar findings to give a wider view. We keep similar findings in a paragraph.

**Comment:** On the point of false positives/negatives... Was the indication of pregnancy suggested by the app itself (i.e., was the app providing pregnancy results?) or was it that the user did a physical pregnancy test and simply shared her disappointment via the app community? If the latter, this point might be better placed in the community support results.

**Reply:** Thank you for your suggestion. We have rephrased the text and given in the below:

“Users initially like to see their pregnancy status in the apps by providing mentioned information like last menstruation date, blood pressure, weight of mother, weakness, vomiting, etc. Based on the information apps give positive or negative results. However, when they found false-negative results based on confirmatory pregnancy tests that impacted their mental stress.”

**Comment:** In the Discussion (p16, line 411-412), you suggest there was a direct link between the usefulness of the apps and the user's overall satisfaction. However, from the analysis method and results presented, it is unclear how you were able to come this such a firm and distinct conclusion. Please revise to be less direct (e.g., 'there may be a link between...') or clarify how such a direct link emerged from qualitative analysis.

**Reply:** Thank you for your suggestion and please consider our unintentional mistakes. We have revised the text as per suggestion. We have given the rephrased text in below:

“Interestingly, useful features and content of the apps impacted the users’ overall satisfaction and positive experiences, sometimes it motivates others to use apps.”

**Comment:** Strengths and limitations... the final sentence needs review for appropriateness and academic language.

**Reply:** Thank you for your suggestion. We have rephrased the required text in the strengths and limitations.

**Comment:** My broad review is reflective of the breadth of revision that I am encouraging before publication may be considered. I hope my above points can be useful in strengthening this manuscript.

**Reply:** Thank you for your inspiring suggestion. We have addressed all of your comments as per your suggestion.
